# Pioneering semi-rigid stability: navigating the female pelvis for enhanced precision in binocular vision guidance

**DOI:** 10.3389/fsurg.2026.1735378

**Published:** 2026-04-28

**Authors:** Tieyuan Sun, Linna Wei, Pan Hu, Xiaolong You, Lubin Liu, Mingbo Liu

**Affiliations:** 1 Department of Obstetrics and Gynecology, Chongqing Health Center for Women and Children, Chongqing, China; 2 Department of Obstetrics and Gynecology, Women and Children's Hospital of Chongqing Medical University, Chongqing, China; 3 Department of Technology Development, Physoft Optical Research Group, Chongqing, China

**Keywords:** binocular vision, pelvic floor, semi-rigid, surface marker, surgical navigation

## Abstract

**Purpose:**

To overcome the surface marker drift issue in semi-rigid pelvic structures and establish a foundation for the binocular vision navigation targeting semi-rigid anatomical structures within the human body.

**Methods:**

The study was conducted at Chongqing health center for women and children form April to June 2024. Surface markers were placed on 20 volunteers with semi-rigid pelvic anatomy. Respiratory and movement-induced displacement data were collected pre- and post-activity. A hybrid approach integrating a loss function and respiratory compensation algorithm was developed for spatial registration correction.

**Results:**

After correction through spatial registration using a mathematical model, the drift range of semi-rigid body surface markers was 0.86 ± 0.11 mm. Specifically, the body surface drift ranges for the left anterior superior iliac spine marker were 0.79 ± 0.12 mm, for the right anterior superior iliac spine marker were 0.85 ± 0.14 mm, and for the pubic symphysis marker were 0.96 ± 0.25 mm. The stability around the umbilicus was relatively poor, with an error range of 1.71 ± 0.91 mm. Among the four markers, three have achieved positioning accuracy meeting the millimeter-level requirements for spatial registration in the current field of medical navigation surgery. The performance complies with the mandated sub-4-millimeter Target Registration Error (TRE) for optical tracking devices in surgical navigation applications.

**Conclusions:**

The first successful mitigation of surface marker drift issues by a mathematical compensation algorithm enabling binocular vision navigation in pelvic floor surgerys, and lays a foundation for future semi-rigid anatomical structure navigations.

## 1 Introduction

Surgical navigation represents a significant research hotspot in medical intelligence. Among these technologies, binocular vision navigation stands out when compared to methods like ultrasound (1) and magnetic navigation (2, 3), owing to its relative maturity, operational stability, high portability, and good accuracy (4, 5).It is expected to become one of the most promising core technologies for surgical navigation. However, it is currently mostly used for surgeries involving rigid structures of the human body, primarily in cranial and large joint surgeries, with only a few reported cases of its use in other body parts (6, 7). The inherent challenge in navigating semi-rigid structures lies in their poor surface stability (8, 9), which is susceptible to respiration and soft tissue deformation, thus failing to meet clinical standards. Addressing this, our research employs the pelvis as a representative model to investigate surface marker stability. As a key outcome of this effort, we have created a novel marker stabilization technique specifically for pelvic floor surgery. By addressing the positional drift caused by tissue elasticity and intraoperative pressure changes, this method enhances navigation reliability in semi-rigid anatomical spaces. These findings establish critical groundwork for implementing precision navigation systems in pelvic floor reconstruction procedures, and. lays a foundation for future navigation targeting semi-rigid anatomical structures.

## 2 Materials and methods

This study utilized patients who visited the outpatient department of Chongqing Maternal and Child Health Care Hospital between April to June 2024. After being fully informed of the research content, body surface stability studies were conducted in the engineering prototype laboratory. The study has been performed in accordance with the ethical guidelines of the 1975 Declaration of Helsinki, and the protocol has been approved by the human research committee at Chongqing Health Center for Women and Children. [Approval No.: (2024) 020]. Informed consent has been obtained from all subjects.

## 3 Experimental design

This study aimed to investigate displacement patterns of cutaneous markers on semi-rigid pelvic structures under different physiological conditions.

Twenty female participants (age range: 26–67 years; BMI: 20.56–29.24 kg/m^2^) underwent sequential biomechanical assessments in the supine position. Cutaneous marker displacement was quantified using a two-phase protocol: Phase I - baseline measurements during quiet tidal respiration; Phase II - post-ambulation measurements obtained after participant repositioning following standardized mobility tasks.

Anatomical marker placement:

• Bilateral anterior superior iliac spines (ASIS):
• Left: Markers 1–4 (2 cm inter-marker spacing)
• Right: Markers 7–10 (2 cm inter-marker spacing)
• Pubic symphysis: Markers 5–6 (superior aspect)
• Umbilical region: Markers 11–14 (periumbilical array)

**Figure 1:**
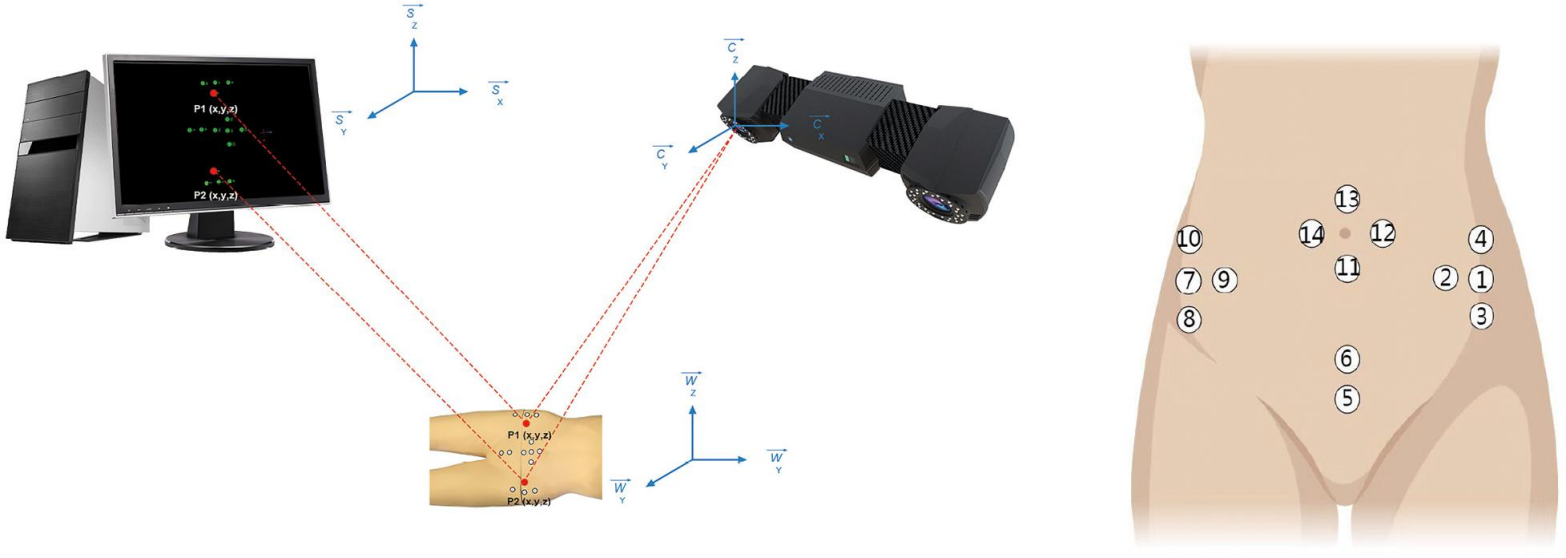
Test mode diagram.

All markers maintained 2 cm transverse spacing within their respective anatomical clusters (Figure 1). Three-dimensional positional data were captured using optoelectronic motion analysis.

## 4 Materials

Equipment: Physoft binocular vision measurement equipment.
Accuracy: *X*-axis 0.15 mm, *Y*-axis 0.15 mm, Z-axis 0.25 mm.
Frame rate: 8 frames per second
Collection time: greater than 30 s.

### 4.1 Experimental environment

Indoor temperature 20–30 degrees Celsius, humidity 10%–90%

### 4.2 Experimental methods

#### 4.2.1 Device configuration

The stereophotogrammetric system (*binocular vision equipment*) was positioned 100–140 cm vertically above the pelvic region, ensuring full visualization of reference markers within the calibrated capture volume.

#### 4.2.2 System calibration

Dual-unit synchronization: Activated primary and secondary motion capture units, verifying inter-device connectivity through dedicated control software.

Dynamic calibration: Positioned a calibration wand with geometrically defined reflective markers within the capture volume to validate spatial resolution (<0.5 mm) and temporal synchronization (sampling rate: 100 Hz).

#### 4.2.3 Participant preparation

**Figure 2:**
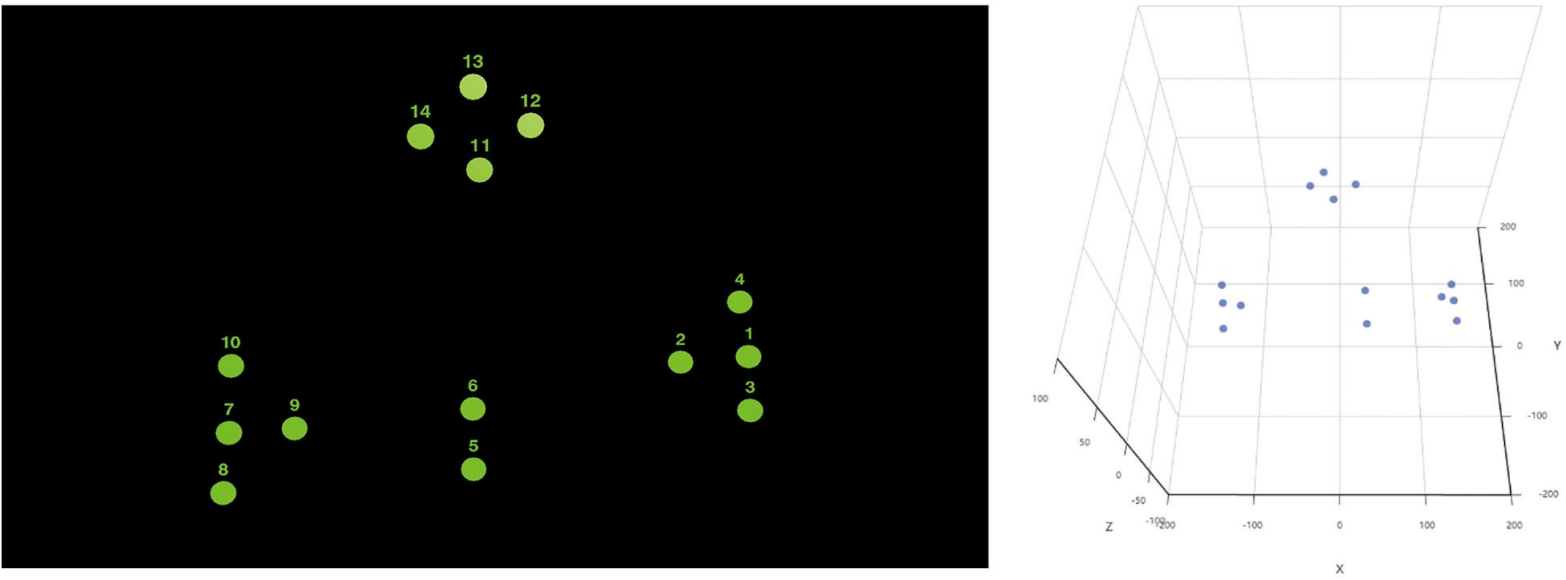
Markers on the surface of a semi-rigid body structure in three-dimensional space.

• Subjects assumed supine positioning with exposed anatomical regions of interest (ROI) beneath the calibrated capture volume.
• ROI alignment was optimized using real-time video feedback to ensure complete marker visibility (Figure 2).

### 4.3 Marker application

Passive retroreflective markers (12.7 mm diameter) were affixed according to predefined anatomical coordinates:

• ASIS clusters: 4 markers/side (mediolateral spacing: 20 mm)
• Pubic symphysis: 2 markers (superoinferior orientation)
• Umbilicus: 4 markers (radial configuration)

#### 4.3.1 Data acquisition protocol

**Baseline phase**: Continuous 15-second capture during quiet respiration (respiratory rate <12 cycles/min).
**Post-mobility phase**: Repeated 15-second acquisition following:
Controlled ambulation (5-meter walk  × 3 repetitions)
Standardized repositioning protocol (supine recumbency within 30 s post-ambulation)

### 4.4 Analysis of non-rigid body matching accuracy

**Figure 3:**
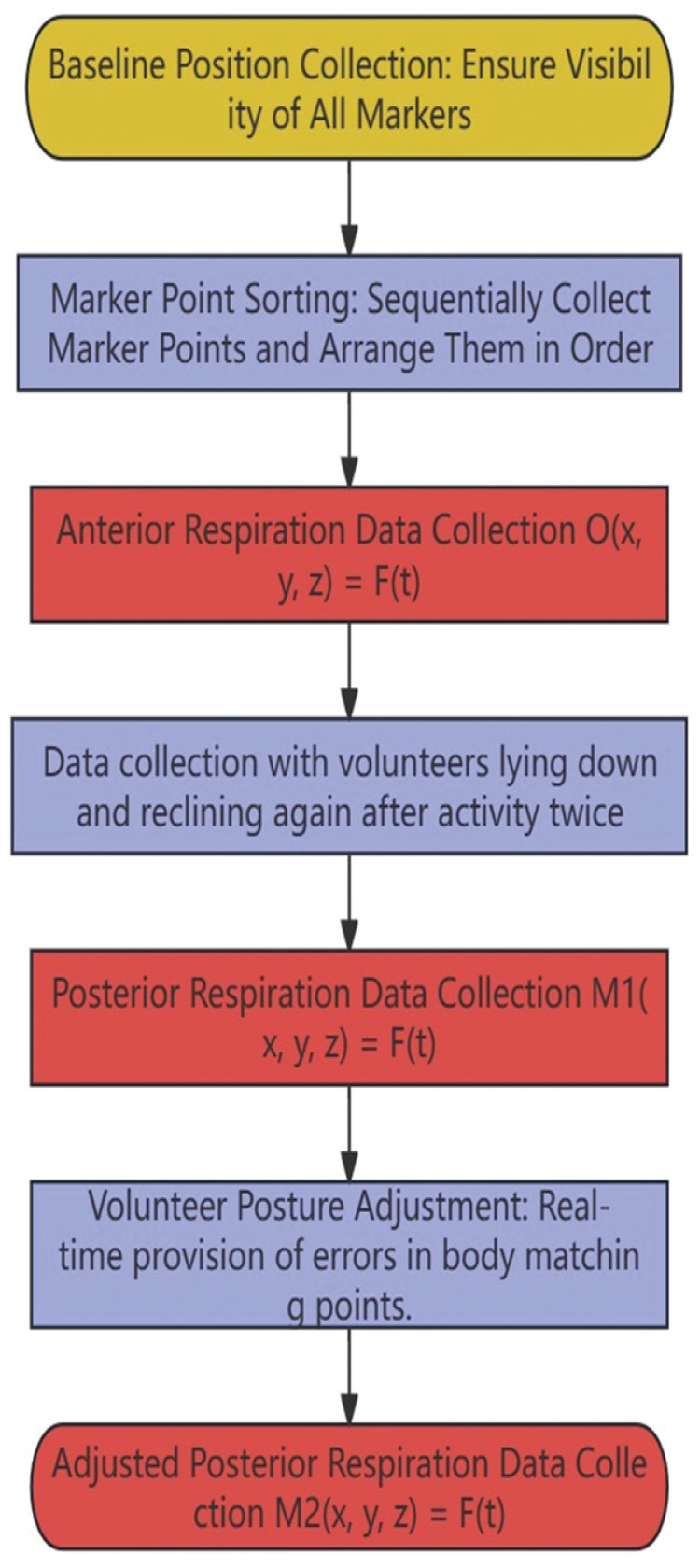
Experimental flowchart.

The experimental Flowchart was shown in Figure 3.

Loss function: $\mathrm{Loss}=\frac{1}{n}\sum_{0}^{n}\;\sqrt{{\left(Ox_{\mathrm{mean}}-Mx_{i}\right)}^{2}+{\left(Oy_{\mathrm{mean}}-My_{i}\right)}^{2}+{\left(Oz_{\mathrm{mean}}-Mz_{i}\right)}^{2}}$

Beline O coordinate system marker points are represented as (Oxi, Oyi, Ozi).

Position-shifted M coordinate system marker points are represented as (Mxi, Myi, Mzi).

#### 4.4.1 Experimental process

During the patient's MRI scan, baseline points O (x, y, z) on the body surface are collected. After the volunteer gets up and moves or with the assistance of experiment personnel, they lie back down at the baseline position, denoted as M1 (x, y, z). Introducing traditional rigid body matching, the correspondence relationship (R, T) between the baseline position and the moved position is solved. Obtaining M1 (x, y, z), real-time display of rigid body matching error index (Loss) is performed using the software system paired with the binocular camera. Adjust the volunteer's posture, focusing on the size of the error index (Loss), to stably improve the accuracy of rigid body matching and obtain M2 (x, y, z). Considering that the marker points on the volunteer's abdomen in the experiment are non-rigid, with flexibility and drift on the skin, and also affected by the coordinate errors caused by the volunteer's own breathing, a respiratory compensation algorithm is applied to eliminate part of the coordinate fluctuation errors caused by breathing. In the process of solving the rigid body matching (R, T) matrix, further optimization is performed at the software code level. The double-variance method is used to filter out a structurally stable set of marker points from the one-to-one corresponding marker points. These points are then used as parameters for solving the rigid body matching matrix. The RANSAC (Random Sample Consensus) algorithm is introduced into the solving process. It iteratively estimates the parameters of a mathematical model from a data set containing “outliers (erroneous points),” resulting in M2 (x, y, z).

### 4.5 Data analysis

Set the duration for dataset collection on the machine. Operate the auxiliary machine and initiate data recording. After completing data collection, retrieve the recorded data from the auxiliary machine using a computer. Analyze the data to obtain experimental results.

#### 4.5.1 Marker classification probability Map

**Figure 4:**
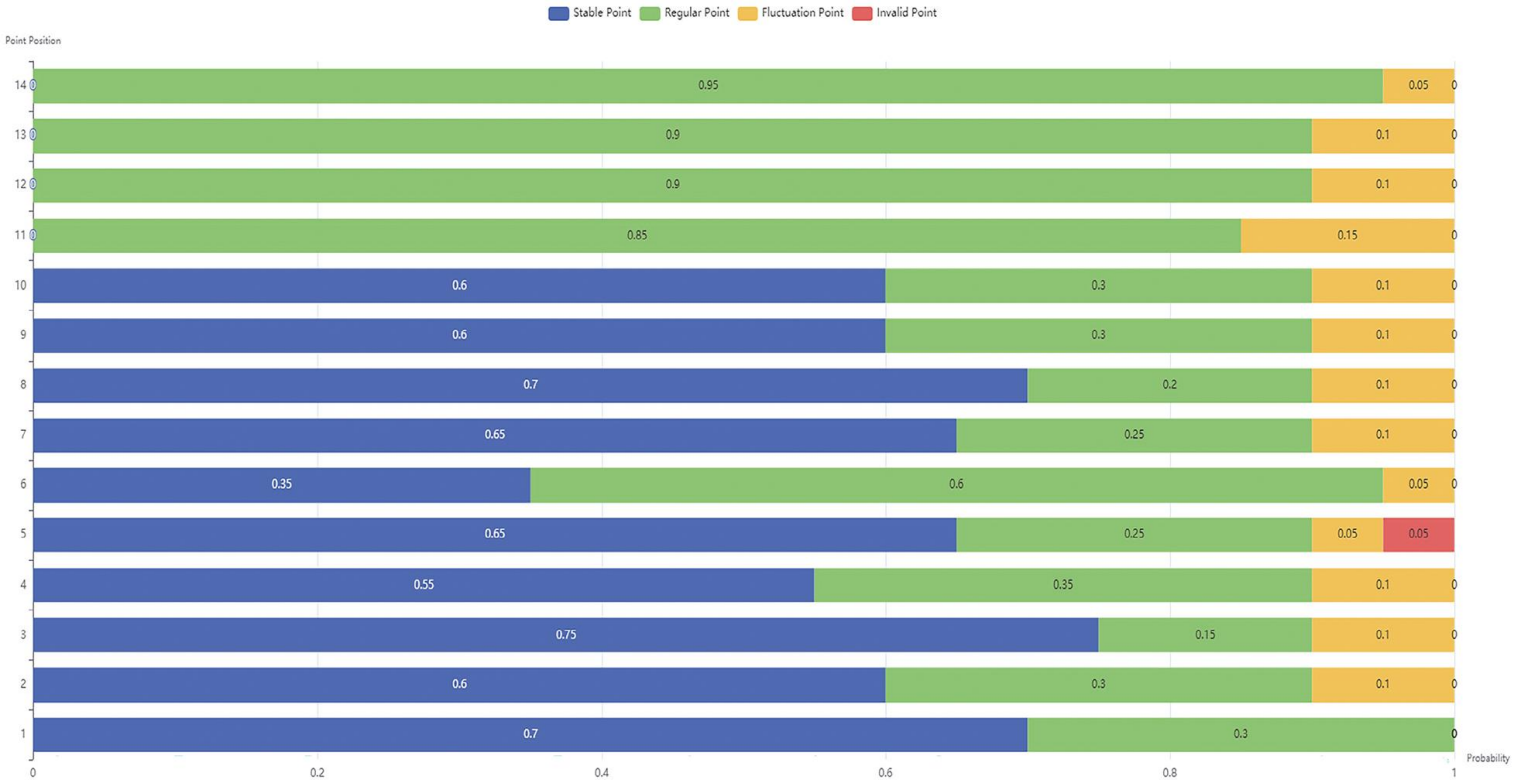
Point correspondence type proportion chart. The vertical axis represents the point number, and the horizontal axis represents the percentage of that point in the population. The probabilities of different types of points are indicated by the same color (for example, Point 2: Probability of stable point in the population is 0.706, regular point is 0.176, fluctuating point is 0.118, and invalid point is 0).

Markers on the body surface have different stabilities in different locations, and markers from different volunteers also exhibit varying stabilities in the same body position (Figure 4). Based on the fluctuations in marker coordinates, regularity, and visibility, markers are classified into four attribute categories.

A: Stable Point: Marker coordinates change by less than 2 mm.

B: Regular Point: Marker coordinates change by more than 2 mm, but the changes exhibit clear periodic patterns.

C: Fluctuating Point: Marker coordinates change by more than 2 mm, with changes lacking regularity and appearing chaotic.

D: Invalid Point: Marker points that cannot be observed normally due to differences in volunteer body shapes or camera angle disparities.

#### 4.5.2 Respiratory compensation algorithms

**Figure 5:**
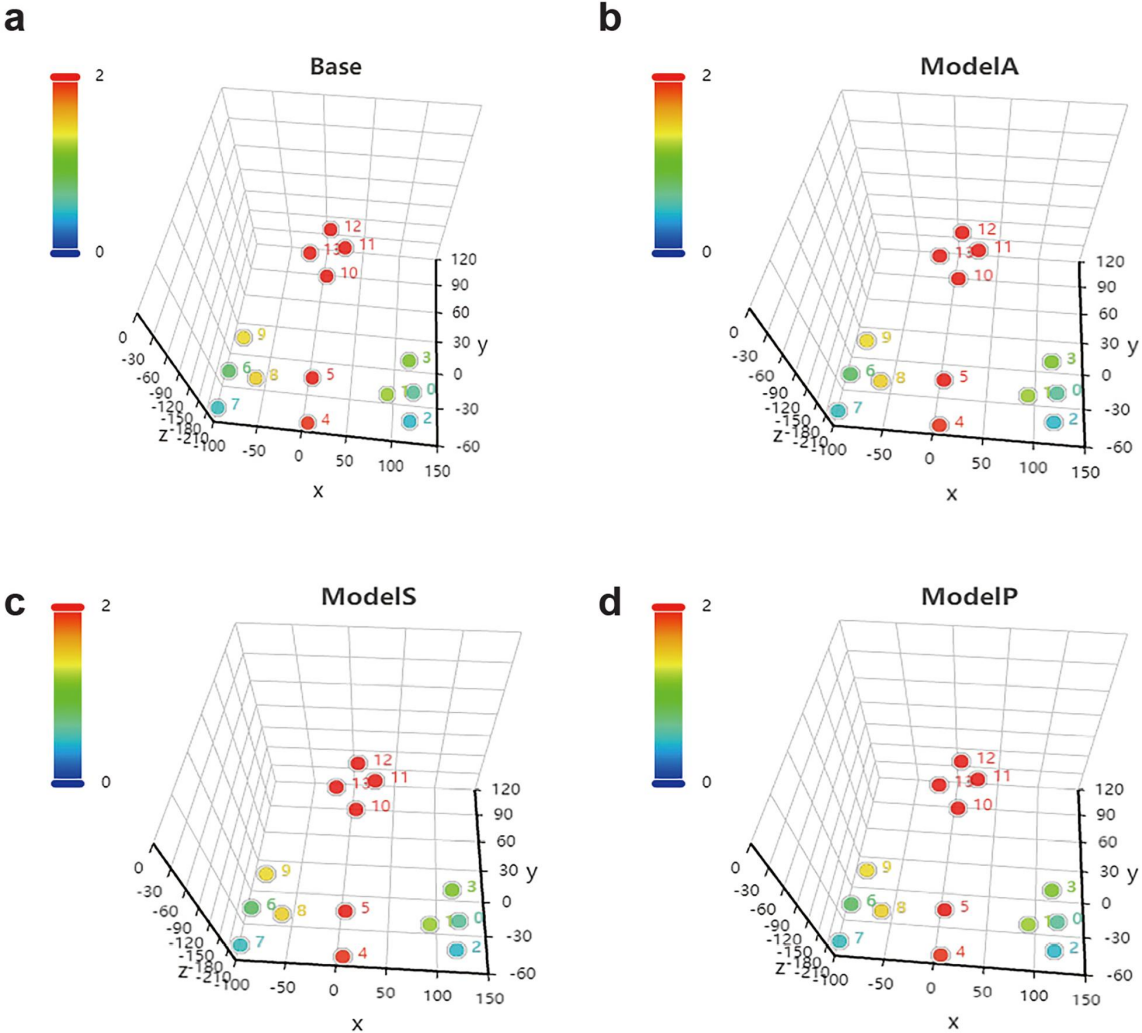
The graph depicts respiratory wave variations processed using a respiratory compensation algorithm. **(a)**: represents the raw data; **(b)**: represents data optimized using mean compensation; **(c)**: represents data optimized using trigonometric compensation; **(d)**: represents data optimized using polynomial compensation. The colored bars on the left side of the graph indicate the amplitude fluctuation range: 0 mm is indicated by blue, 1 mm by green, and values exceeding 2 mm are represented by red.

The graph depicts respiratory wave variations processed using a respiratory compensation algorithm (Figure 5). Decomposing the motion into three-dimensional (x, y, z) components, trigonometric functions—being periodic—can model respiratory patterns. Their amplitude directly represents the magnitude of respiratory motion. Polynomial functions can also model complex respiratory patterns by adjusting the polynomial order, enabling subsequent statistical extraction of breathing cycles and respiratory features. Understanding respiratory motion patterns allows for the mitigation of point drift and fluctuations caused by breathing motion. Spectral Subtraction is employed to subtract the fitted respiratory spectrum from the acquired respiratory spectrum, thereby eliminating the impact of respiratory fluctuations.

a) Mean Compensation: Given respiratory data within a time sliding window, calculate the fluctuation mean of marker points. Given upper and lower limits of fluctuation, use them to correct subsequent respiratory marker point data.
b) Polynomial Compensation: Use a polynomial to fit the respiratory fluctuation curve. Reverse the polynomial curve, overlay it with the respiratory curve, and compensate for the error impact of respiration.
c) Trigonometric Compensation: Respiration follows a regular pattern and satisfies a sinusoidal wave curve. Use a trigonometric sine function to fit the respiratory fluctuation curve. Reverse the fitted curve, overlay it with the respiratory curve, and compensate for the error impact of respiration.

## 5 Results

The surface stability of semi-rigid structures can be improved by introducing a Loss function to enhance precision.

**Figure 6:**
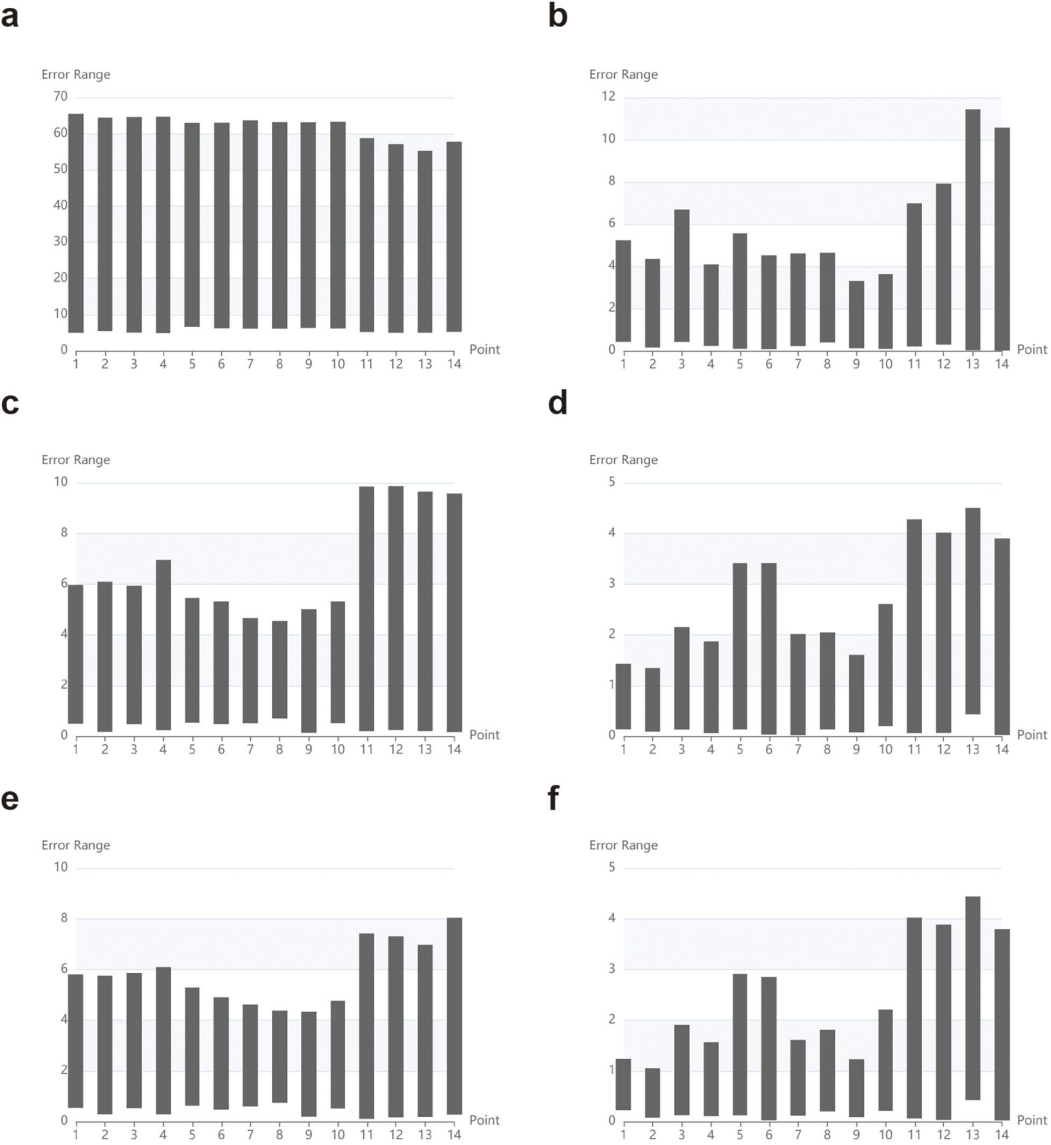
Error K, line chart. **(a)**: after breathing; **(b)**: after breathing + rigid body matching; **(c)**: after breathing + adjust posture; **(d)**: after breathing + adjust posture; **e**: after breathing + adjust posture (breathing compensation); **(f)**: after breathing + adjust posture + rigid body matching (breathing compensation).

In the experiment, 14 surface markers on the pelvic surface were collected to establish coordinate system M1^、^(x, y, z) (as shown in Figures 6a,b). During the spatial registration process, it was observed that the surface marker drift ranged from 3.2 to 11.4 mm, primarily attributed to soft tissue deformation caused by coverage issues. The precision of points in M1, failed to meet the requirements for surgical navigation.

To address this, we introduced fine adjustments to the posture of volunteers during data collection to ensure stable point recognition. Innovatively, we incorporated a Loss function into the semi-rigid structure framework, which proved effective in improving surface stability. This led to the creation of coordinate system M2 (x, y, z) (as shown in Figures 6c,d).

The surface drift measurements for specific markers were as follows: the left anterior superior iliac spine marker exhibited a drift of 0.82 ± 0.13 mm, the right anterior superior iliac spine marker showed a drift of 0.88 ± 0.17 mm, the pubic symphysis marker demonstrated a drift of 1.67 ± 0.36 mm, and the umbilical marker had a drift of 1.74 ± 0.91 mm. These results highlight the effectiveness of the Loss function in enhancing surface stability for semi-rigid structures.

The iterative approach of the mathematical model can address the coordinate errors of marker points induced by respiratory movement

While the Loss function reduces the drift range of surface markers in semi-rigid structures, coordinate errors in skin-based marker points persist due to factors such as flexibility, drift, and respiratory movement. Since respiratory movement exhibits non-linear behavior, we employed an iterative mathematical model to estimate non-linear parameters. The RANSAC algorithm tackles the pose estimation problem (R, T). (**Parameter Configuration:** 1. Number of Iterations: 100. This value represents a robust choice for most scenarios. 2. Outlier Threshold: Adaptively determined based on statistical analysis of multiple volunteers’ data, accounting for point errors induced by respiratory fluctuations. Set to: Mean Error + 1.5 × Standard Deviation. 3. Confidence: 0.99. 4. Nonlinear Refinement: Enabled to improve final accuracy). Pose estimation is a critical step in the experiment. During the optimization process, the RANSAC algorithm was introduced to enhance the robustness of the solution for baseline position and movement (R, T) values, resulting in the refined coordinate system M2、(x, y, z) (Figures 6e,f).

Through optimization using a mathematical fitting model and compensatory algorithm, the drift range of semi-rigid body surface markers was reduced to 0.86 ± 0.11 mm. Specifically, the surface drift measurements for individual markers were as follows:

• Left anterior superior iliac spine marker: 0.79 ± 0.12 mm

• Right anterior superior iliac spine marker: 0.85 ± 0.14 mm

• Pubic symphysis marker: 0.96 ± 0.25 mm

• Umbilical marker: 1.71 ± 0.91 mm (notably less stable compared to other markers).

These results demonstrate the effectiveness of the iterative mathematical model and compensatory algorithm in mitigating coordinate errors induced by respiratory movement, while maintaining surface stability in semi-rigid structures.

## 6 Discussion

Recently, binocular vision navigation has shown promise for its high precision (10), yet existing systems are confined to rigid structures like the skull or joints (11, 12) and cannot adapt to dynamic tissue shifts in semi-rigid pelvic settings (7, 13). The core obstacle in surgical navigation for semi-rigid anatomy is that physiological deformations during the procedure lead to substantial spatial registration errors. Therefore, overcoming the surface drift issue in semi-rigid structures becomes a crucial key to unlocking its potential. Given the unique semi-rigid anatomy of the pelvis and the high risks associated with blind puncture techniques in pelvic floor reconstruction (14–16), this study selects the female pelvis as a research model to explore safer and more accurate surgical solutions. Visualizing these blind procedures could transform pelvic floor surgery by enhancing targeting accuracy. Previous literature (17) has explored the spatial registration issues between surface markers and pelvic internal structures using fresh-frozen cadavers. The study simulated the impact of changes in body position from CT examination to surgery on surface stability. Through compensating for sacral tilt, the smallest recorded surface drift error was 1.9 mm (in a non-pneumoperitoneum state). However, since the specimens were stiff cadavers without respiratory movement, the actual error in surgery could be larger. In another study, during rectal cancer surgery using semi-rigid structure spatial registration, the reported error range in assisted surgical navigation was within ±4 mm (6, 18–20). These studies reports the smallest error to date in semi-rigid structure navigation. Nevertheless, the achieved accuracy remains insufficient to meet clinical requirements. Therefore, if a compensatory algorithm for data under non-invasive marking could be used to correct surface drift issues, it would lay the foundation for the practicality and widespread use of navigation in the semi-rigid structures of the human body. In this study, the research team made the first attempt in a semi-rigid pelvic structure, verifying the feasibility and superiority of the technology through surface marker and stability experiments. Through mathematical compensation algorithms, the data error from surface drift was reduced to within 1 mm, thereby approaching the stringent sub-0.5 mm spatial registration standard required for optical navigation of rigid anatomical structures (21).

Overcoming surface drift in semi-rigid structures involves two steps: the first step addresses the non-rigid structure spatial matching problem, and the second step focuses on solving the respiratory motion compensation problem. Firstly, the key issue in non-rigid structure spatial matching refers to the drift of surface markers after movement. In this experiment, the patient's supine position, post-MRI examination, walking, and finally lying on the operating table were simulated to observe the drift error of surface markers. According to rigid body matching data statistics, the coordinate system M1, composed of 14 pelvic markers showed a surface marker drift range of 3.2–11.4 mm during spatial registration. This was mainly due to drift caused by soft tissue coverage issues, and it was observed that the lateral points of the anterior superior iliac spine were partially lost due to occlusion. The precision of points in M1、couldn't meet the requirements for surgical navigation. Therefore, in the experiment, fine adjustments to the posture collected by fixing volunteers were made to ensure stable point recognition. We innovatively introduced the use of a Loss function in semi-rigid structures, which proved helpful in improving the stability on the surface of semi-rigid structures, resulting in coordinates M2 (x, y, z). The surface drift range for pelvic markers (left anterior superior iliac spine, right anterior superior iliac spine, and pubic symphysis) was between 0.02–3.40 mm. However, such a range of surface marker drift errors might lead to larger spatial registration errors in the internal data of semi-rigid structures. By analyzing, it was found that pelvic surface markers not only exhibit flexibility and drift on the skin but also experience coordinate errors induced by respiratory movement. Therefore, we proposed a solution to overcome the second problem: using a respiratory compensation algorithm to eliminate some of the coordinate fluctuation errors caused by breathing. In previous studies, coordinate system calibration errors were often corrected using calibration boards. However, in semi-rigid structures, respiratory motion exhibits non-linear behavior. Hence, we used the iterative approach of a mathematical model to estimate non-linear parameters and incorporated the RANSAC algorithm during the solving process to improve the robustness of the solution for baseline position and movement (R, T) values, resulting in M2 (x, y, z). Through mathematical fitting model optimization and compensatory algorithm, it was found that the stability of the marking point at the navel was poor, with an error range of 1.71 ± 0.91 mm. This is mainly because volunteers may exhibit abdominal breathing, thoracic breathing, or even both, so we exclude the use of this marker point. However, the drift range precision of the surface marking points on the other three pelvic semi-rigid body structures is high. The drift range on the surface of the left anterior superior iliac spine marker point is 0.79 ± 0.12 mm, the drift range on the surface of the right anterior superior iliac spine marker point is 0.85 ± 0.14 mm, and the drift range on the surface of the pubic symphysis marker point is 0.96 ± 0.25 mm. This is better than the drift range of surface optical surgical navigation marker points reported in previous literature, which ranged from 1.9 to 4 mm (6, 17), meeting the requirement of marker point error range within 2 mm for optical surgical navigation spatial registration (22). This fully validates that the drift of surface optical marker points in the human semi-rigid structure is a solvable problem and technically proves the feasibility of improving spatial registration in semi-rigid structures, providing reference for later semi-rigid body surgical navigation.

Therefore, we focused our research on the pelvis as a semi-rigid structure. By utilizing surface landmarks of anatomical structures and employing mathematical compensation methods, we successfully addressed the technical challenges of overcoming surface drift in semi-rigid structures. This achievement reached millimeter-level precision, addressing the significant issue of surface marker drift caused by respiratory movements and the coverage of pelvic surface soft tissues in different body positions. This breakthrough in overcoming the technical challenges of surface drift in semi-rigid structures provides a theoretical foundation for reducing spatial registration errors in later stages. The application prospects for this breakthrough are immense, particularly in the field of human surgical navigation spatial registration. In the future, it can be applied to chest surgery, liver and kidney punctures, and fixed-point radiotherapy similar to semi-rigid structures. While this research has successfully addressed surface drift issues in pelvic surgery navigation in the supine position, we encountered challenges related to pelvic displacement due to hip joint movement during the experimental process. Hip joint movement can cause irregular displacement of pelvic surface markers, which cannot be corrected through mathematical models. For surgeries requiring lithotomy positions, our next step involves using a body model or transferring pelvic stable targets to externally fixed points to overcome the decrease in accuracy caused by hip joint movement. After transferring the target, the stability of the semi-rigid pelvis relative to the fixed target on the operating table is higher, thus achieving semi-rigid structure navigation in lithotomy positions.

This study overcomes the issues of stability and accuracy in the three-dimensional space of semi-rigid structures through the combination of multiple-point marking of surface key points and mathematical compensation algorithms. The resolution of surface drift opens up the possibility of breaking through the bottleneck of binocular vision technology in surgical navigation in pelvic floor surgery and other semi-rigid structures. In the future, surgeries within semi-rigid structures such as pelvic organs could utilize surface localization to achieve precise positioning of internal stable structures, thereby enhancing the accuracy of surgical navigation within these semi-rigid structures.

## 7 Conclusions

By selecting surface markers with smaller errors and integrating multi-point markers with mathematical compensation algorithms, surface drift in the semi-rigid pelvic region can be effectively addressed. This approach enables the application of binocular vision technology in surgical navigation under semi-rigid structures. The combination of multi-point marker selection and mathematical compensation algorithms significantly reduces surface drift in the semi-rigid pelvic region, thereby achieving millimeter-level precision for binocular vision surgical navigation.

## Data Availability

The datasets presented in this study can be found in online repositories. The names of the repository/repositories and accession number(s) can be found in the article/Supplementary Material.
